# Patient-Reported Outcome on Quality of Life and Pain after Revision Arthroplasty for Periprosthetic Joint Infection: A Cross-Sectional Study

**DOI:** 10.3390/jcm11237182

**Published:** 2022-12-02

**Authors:** Chaofan Zhang, Ziyu Liu, Yunzhi Lin, Yuanqing Cai, Xuehui Zhang, Zida Huang, Ying Huang, Wenbo Li, Xinyu Fang, Wenming Zhang

**Affiliations:** 1 Department of Orthopaedic Surgery, The First Affiliated Hospital, Fujian Medical University, Fuzhou 350005, China; 2 Department of Orthopaedic Surgery, National Regional Medical Center, Binhai Campus of the First Affiliated Hospital, Fujian Medical University, Fuzhou 350212, China; 3 Fujian Provincial Institute of Orthopedics, The First Affiliated Hospital, Fujian Medical University, Fuzhou 350005, China; 4 Department of Stomatology, The First Affiliated Hospital, Fujian Medical University, Fuzhou 350005, China; 5 Department of Stomatology, National Regional Medical Center, Binhai Campus of the First Affiliated Hospital, Fujian Medical University, Fuzhou 350212, China; 6 Department of Orthopaedics, The Second Affiliated Hospital of Xi’an Jiaotong University, Xi’an 710006, China; 7 School of Health Management, Fujian Medical University, Fuzhou 350005, China

**Keywords:** periprosthetic joint infection (PJI), cross-sectional study, questionnaire, 36-item Short Form Health Survey (SF-36), McGill Pain Questionnaire (MPQ)

## Abstract

This study aims to explore the quality of life (QOL) and pain after revision surgery for periprosthetic joint infection (PJI) based on patients’ reported outcomes. A cross-sectional questionnaire survey was conducted and 137 valid responses were included (response rate 64.0%). A total of 42 patients underwent debridement with implant retention (DAIR), 31 underwent one-stage revision, and 64 underwent two-stage revision. The average overall SF-36 score was 70.3. The DAIR group had significantly higher SF-36 than the two-stage revision group (*p* = 0.01). There was no significant difference between the one-stage revision group and the other two groups. A total of 74.5% of patients reported pain with an average McGill Pain Questionnaire (MPQ) score of 8.6. There was no significant difference in the MPQ scores among the three groups. Simple linear regression analyses demonstrated that higher preoperative PMN%, VAS, and shorter hospital stay were associated with pain (adjusted R^2^ = 4%, *p* = 0.020; adjusted R^2^ = 2.1%, *p* = 0.048; adjusted R^2^ = 2.1%, *p* = 0.049; respectively). We concluded that the overall QOL of patients after revision surgery for PJI is generally satisfactory. Persistent pain is prevalent, but the severity was mostly mild. Preoperative PMN%, VAS, and hospital stay were associated with postoperative pain.

## 1. Introduction

Joint replacement surgery has been considered to be ‘the operation of the century’ [1]. It was estimated that internationally the number of total hip arthroplasty (THAs) is projected to increase by 170% by the year 2030 [2]. For total knee arthroplasty (TKA), statistics have shown the number of TKAs in the United States, which already has the highest number of knee arthroplasties in the world, is expected to increase by 143% by 2050 [3]. In China, because of an aging population and improving economic conditions, the demand for joint arthroplasty has dramatically increased [4]. In a recent study, about 50,000 hip or knee arthroplasties are annually performed in China and this number is increasing every year by ~15% [4].

Although joint replacement surgery is widely performed, infection after surgery, also known as periprosthetic joint infection (PJI), remains a catastrophic complication for patients following arthroplasty. Although the incidence of PJI is low (about 2% for hip replacements and 2.4% for knee replacements), the repercussions are severe when they do occur [5]. Patients usually need to undergo multiple additional revision surgeries, which are accompanied by high morbidity and mortality [6]. After surgery, patients may suffer from persistent chronic pain and dysfunction in varying degrees, which places a heavy burden on society and the economy [7]. With the increase in the number of joint replacements, the number of PJIs is also showing a significant increasing trend. It is predicted that there will be 100,000 cases of revision surgery caused by PJI before 2030 [6].

Treatment of PJI is based on several factors, including the timing of symptoms, the infecting organism, and the health of the host, and it involves techniques such as irrigation and debridement with implant retention (DAIR) for acute infections and two-stage exchange arthroplasty or single-stage exchange arthroplasty for the treatment of chronic PJI [6]. Regardless of the revision strategy, patients usually have significant residual pain and dysfunction due to the need for thorough debridement [8,9]. The impact of two-stage revision surgery on patients might be greater than that of DAIR or one-stage revision because the waiting time between the two stages means a longer period of immobility and greater psychological pressure [8]. Unfortunately, there is a paucity of research on the prevalence, risk factors of pain, and quality of life (QOL) following revision surgery for PJI.

The current methods for assessing the results of joint replacement surgery are based mainly on the clinical signs and symptoms, physical examination, and radiographic evaluation. The routine clinical examinations do not take into account the QOL following revision surgeries [10]. Currently, there are only a few investigations on the functional recovery, QOL, and pain improvement of patients after PJI revision surgery. Specifically, very limited information is available regarding the patient-reported health-related quality of life (HRQOL) and pain following treatment for PJI. Sabah et al. have explored changes in patient-reported outcome measures (PROMs) after revision TKA (rTKA) in 10,727 patients and found that two-thirds of patients experienced a meaningful improvement in joint function after rTKA. However, there was a high frequency of patient-reported complications [11]. Walter et al. have investigated the long-term QOL in 36 patients with achieved eradication of infection after knee PJI. They concluded that PJI patients still suffer from significantly lower quality of life compared with normative data, even years after surgically successful treatment by either DAIR, one/two-stage revision exchange, or arthrodesis [12]. Another study compared the outcome of 52 knee arthrodesis with 52 hinged TKA in patients suffering from knee PJI and showed arthrodesis using a modular intramedullary nail is an alternative for limb salvage, which reduced pain and increased quality of life (SF-12 score) and everyday mobility, when revision TKA is not an option [13]. PROMs and patient-reported experience measures (PREMs) provide a standardized assessment of the patients’ health status or experience with health care directly from the patient, which is also essential for clinicians to understand the impact a treatment has on patients’ lives [14].

Therefore, the purpose of this study was: (1) to investigate QOL and pain improvement of patients after PJI revision surgery; (2) to compare the differences in patients’ QOL and pain improvement after different treatment strategies; (3) to explore the potential factors affecting patients’ QOL and pain after revision surgery for PJI. Such information may influence current practice to stratify risk factors and counseling of patients before surgical procedures and may direct future research toward the mechanisms, prevention, and treatments for patients who are at high risk of persistent postsurgical pain after revision surgery for PJI.

## 2. Materials and Methods

### 2.1. Participants

This study is a cross-sectional observational investigation using a web-based questionnaire survey conducted by a single center. The study has been approved by the ethics committee of the First Affiliated Hospital of Fujian Medical University ((2022) 218, dated 27 April 2022). Participants were informed that their participation was voluntary. Online informed consent was obtained from each participant. Patients who were diagnosed with PJI and underwent revision surgery (including DAIR, one-stage revision, and two-stage revision) in our department from January 2007 to June 2022 were retrospectively reviewed. Demographic information of the patients (age, gender, body mass index (BMI), surgical site, smoking history, drinking history) and the perioperative data (including the number of previous operations in the same joint, American Society of Anaesthesiologists (ASA) score, intraoperative blood loss, length of hospital stay, and follow-up time) were traced from the electronic medical records. The number of previous operations in the same joint includes the primary arthroplasty. For two-stage revision, intraoperative blood loss and length of hospital stay were defined as the sum of 2 hospital visits.

### 2.2. Inclusion and Exclusion Criteria

#### 2.2.1. Inclusion Criteria

(1) Patients diagnosed with PJI according to the 2018 consensus meeting diagnostic criteria [15]; (2) received revision surgery, including DAIR, one-stage revision, and two-stage revision; (3) follow-up time since the last operation was more than 1 year.

#### 2.2.2. Exclusion Criteria

(1) Patients who gave up treatment, chose conservative treatment with oral antibiotic suppression, or simply underwent single debridement surgery; (2) the follow-up time since the last operation is less than 1 year; (3) dead patients or patients who were lost to follow-up.

### 2.3. Questionnaire

The complete Rand 36-Item Short Form Health Survey (SF-36) was incorporated into the questionnaires to assess the QOL after revision surgery [16]. SF-36 was a short-form functional health and well-being survey; it is well documented and has been used and validated in orthopedic patients, including those who received THA and TKA [17]. The scores of 8 independent scales were calculated, with 2 dimensions (physical health and mental health) and total SF-36 results. Scores in the SF-36 were converted to a 0 to 100 scale (0 = worst and 100 = best). This conversion has been previously used after THA and TKA [17,18]. 

The short-form McGill Pain Questionnaire (MPQ) was used for the description of pain [19]. The MPQ consists of 15 adjectives describing sensory, affective, and evaluative aspects of pain experience. Patients rate the adjectives (Pain Rating Index (PRI)) that best describe their current pain with a four-point scale, with end points of 0 (none) and 3 (severe) and a total score of 45. Patients also rate their present pain intensity on a visual analogue scale (VAS) and the overall intensity of their total pain experience on a numerical rating scale, with the endpoints 0 (no pain) and 5 (excruciating pain) [20]. 

The questionnaires were originally in English and were then translated into Chinese. Local experts validated the content of the questionnaire, after which it was pilot-tested. The SF-36 questionnaire showed a reliability (Cronbach’s alpha) of 0.829 and a validity (Kaiser–Meyer–Olkin test coefficient, KMO) of 0.768. For the MPQ questionnaire, a Cronbach’s alpha of 0.890 and a KMO of 0.782 was achieved. The returned questionnaires were reviewed carefully and the invalid or incomplete questionnaires were removed. After completion, surveys were collected and responses were manually entered into a password-protected database that was accessible only to the IRB-approved study personnel. The English version of the questionnaire is shown in Supplementary Materials.

### 2.4. Statistical Analysis

The SF-36 scores and MPQ pain scores of all patients were summarized, calculated, and further compared among the three surgical groups. Continuous variables (including age, BMI, number of previous operations in the same joint, ASA score, intraoperative blood loss, length of hospital stay, follow-up time, SF-36 score and subscale scores, MPQ scores and subscale scores, and VAS score) were presented as mean (standard deviation (SD)). Categorical variables (including gender, surgical site, smoking history, and drinking history) were presented as frequency. Normality testing of data was performed using the Shapiro–Wilk test, and homogeneity of variance testing was performed using the Levene test. Differences in quantitative data among the three groups were assessed using one-way ANOVA, followed by the Tukey–Kramer HSD post hoc test. Bivariate correlation analysis was performed to investigate the association between the demographic information, perioperative indicators, SF-36 score, and MPQ score. Simple linear regression analysis was performed to further explore the risk factors for the SF-36 score and MPQ score. All statistical analyses were performed using SPSS, version 20.0 (IBM Corporation, Armonk, NY, USA). The level of significance was set at *p* < 0.05.

## 3. Results

### 3.1. Demographic Information

**Figure 1:**
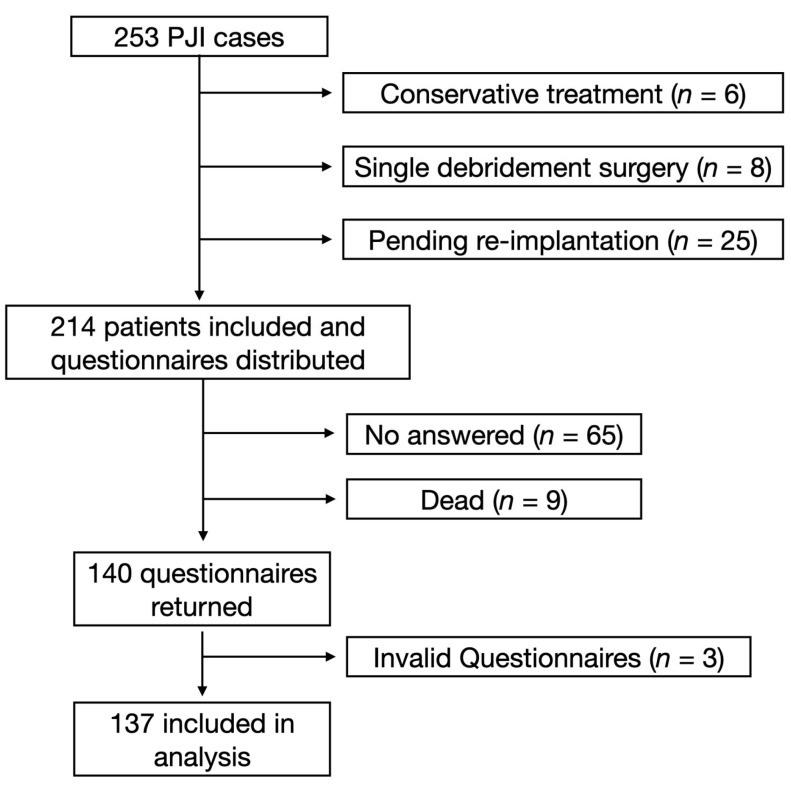
Flow diagram of the current study.

From January 2007 to June 2021, a total of 253 patients were diagnosed with PJI and treated in our department. Among them, 214 patients who met the inclusion criteria were included in the study and 39 patients were excluded (6 patients were treated with conservative treatment with antibiotics only, 8 patients were treated with single debridement only, and 25 patients received the two-stage revision but the second-stage reimplantation was not performed). A total of 214 questionnaires were distributed. Sixty-five patients did not respond to the questionnaire, and nine patients died due to non-PJI-related diseases, leaving 140 responses returned. After further review of the responses, 3 invalid responses were excluded and 137 valid responses were ultimately included. The response rate was 64.0% (137/214). All surgeries were performed by the same senior surgeon. Patient demographic information (including age, sex, BMI, surgical site, smoking history, and drinking history) was traced from the medical record system and questionnaire data were extracted for further statistical analysis. The research flow diagram is shown in Figure 1.

The average age of the 137 patients was 67.6 (9.8) years, including 64 males and 73 females, with an average BMI of 24.1 (3.1). There were 74 cases of infection after THA and 63 cases of infection after TKA. A total of 42 patients underwent DAIR surgery, 31 patients underwent one-stage revision, and 64 patients underwent two-stage revision surgery. The average follow-up time was 35.3 (20.9) months. The demographic information is shown in Table 1.

**Figure 2:**
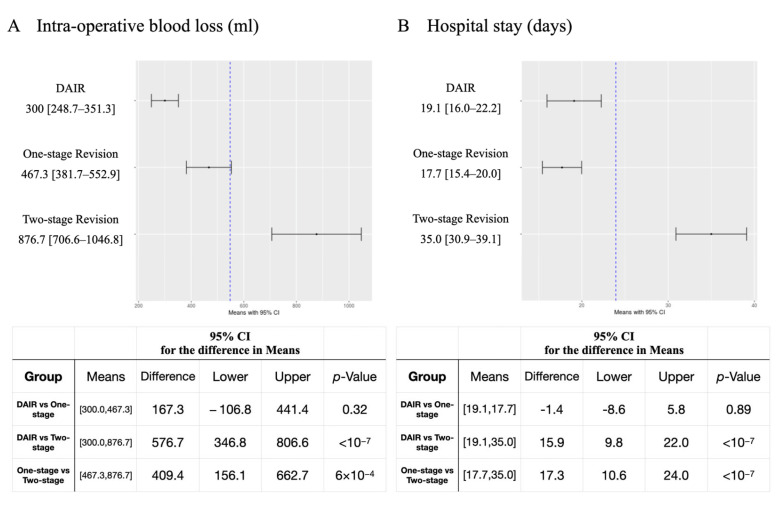
Tukey–Kramer HSD post hoc test of intraoperative blood loss and hospital stay. (**A**) Intraoperative blood loss, (**B**) hospital stay.

Statistical analysis showed significant differences among the three groups in the number of previous operations in the same joint (*p* = 0.01), intraoperative blood loss (*p* < 0.0001), and the length of hospital stay (*p* < 0.0001). Further post hoc test results showed that the number of previous operations in the same joint in patients with two-stage revision was significantly higher than that in the DAIR group (mean difference 0.46, 95% Confidence Interval (CI) 0.07–0.86; Hedges’ g =0.54, 95% CI 0.15–0.94; *p* = 0.02). For intraoperative blood loss, the two-stage revision group was significantly higher than the DAIR group (mean difference 576.7, 95% CI 346.8–806.5; Hedges’ g =1.06, 95% CI 0.65–1.48; *p* < 0.0001), and the one-stage revision group (mean difference 409.4, 95% CI 156.2–662.7; Hedges’ g = 0.71, 95% CI 0.27–1.15; *p* < 0.0001) (Figure 2A). For the length of hospital stay, the two-stage revision group was significantly higher than the DAIR group (mean difference 15.9, 95% CI 9.8–22.0, Hedges’ g = 1.11, 95% CI 0.70–1.53; *p* < 0.0001), and the one-stage revision group (mean difference 17.3, 95% CI 10.6-24.0; Hedges’ g = 1.23, 95% CI 0.77–1.70; *p* < 0.0001) (Figure 2B). There were no significant differences regarding patients’ age, sex, BMI, surgical site, history of smoking, history of alcohol drinking, ASA score, and follow-up time. (Table 1)

### 3.2. QOL after Revision Surgery for PJI

All patients completed the SF-36 questionnaires. The overall score was 70.3 (15.6), in which the scores of physical health (PH) and mental health (MH) dimensions were 66.8 (18.3) and 72.0 (16.5), respectively. The scores of the eight subscales and ‘Health change’ are shown in Table 2. In general, patients’ QOL after receiving revision surgery for PJI was acceptable (70.3 of 100.0).

As shown in Table 2, there were significant differences among the DAIR, one-stage revision, and two-stage revision groups on the SF-36 overall score, Dimension A (physical health (PH)) score, Scale 1 (physical functioning (PF)) score, and Scale 2 (role limitations due to physical health) score (*p* = 0.02, *p* = 0.04, *p* = 0.005, and *p* = 0.02, respectively). Further post hoc test analyses showed that for the SF-36 overall score, PH score, and Scale 2 score, the values in patients with DAIR were statistically significantly higher than those in the two-stage revision group: (mean difference 8.49, 95% CI 1.30–15.70; Hedges’ g = 0.54, 95% CI 0.14–0.94; *p* = 0.01) for SF-36 overall score, (mean difference 8.97, 95% CI 0.51–17.4; Hedges’ g = 0.49, 95% CI 0.09–0.88; *p* = 0.03) for PH score, and (mean difference 22.0, 95% CI 3.08–41.0; Hedges’ g = 0.53, 95% CI 0.14–0.93; *p* = 0.02) for Scale 2 score, respectively. For the Scale 1 score, the value in patients with DAIR was statistically significantly higher than that in the two-stage revision group (mean difference 12.4, 95% CI 2.24–22.5; Hedges’ g = 0.56, 95% CI 0.16–0.96; *p* = 0.01) and the value in patients with one-stage revision was also significantly higher than that in the two-stage revision group (mean difference 12.1, 95% CI 0.98–23.3; Hedges’ g = 0.52, 95% CI 0.09–0.96; *p* = 0.03). This result suggests that patients with DAIR had significantly higher QOL and physical functioning than the two-stage revision surgery. However, it should be acknowledged that the standardized effect size for all the differences was low as represented by the Hedges’ g and lower limit on d values, especially for the PH score and Scale 1 score. However, there was no significant difference in the Dimension B (mental health (MH)) score, the scores of the other six subscales, and the health change score among the three groups.

### 3.3. Pain after Revision Surgery for PJI

All patients completed the McGill Pain Score questionnaires. The total McGill pain score, sensory subscore, affective subscore, VAS score, present pain intensity (PPI), and change of preoperative and current VAS score are shown in Table 3. Overall, we observed prevalent persistent pain (with VAS > 0) in patients receiving revision surgery for PJI, with 74.5% (102/137) of pain after revision at a mean follow-up time of 35.3 months. In general, the pain of patients receiving revision surgery for PJI was mild (average total MPQ score of 8.6 of 45). However, there was no significant difference in the pain indicators among the three groups. The change of VAS was also not significantly different among the three groups (3.9 (3.2) in DAIR vs. 4.7 (2.6) in one-stage revision vs. 3.2 (3.5) in two-stage revision, *p* = 0.09).

### 3.4. Potential Risk Factors for QOL and Pain after Revision Surgery for PJI

The results of the bivariate correlation analysis between the demographic information, perioperative indicators and SF-36 score, and MPQ score are presented in Table 4. There was no significant association between SF-36 and age, sex, BMI, surgical site, number of previous operations in the same joint, ASA score, inflammatory makers (including C reactive protein (CRP), erythrocyte sedimentation rate (ESR), synovial fluid white blood cell count (WBC), percentage of polymorphonuclear white blood cell (PMN%)), preoperative VAS score, intraoperative blood loss, and hospital stay.

**Figure 3:**
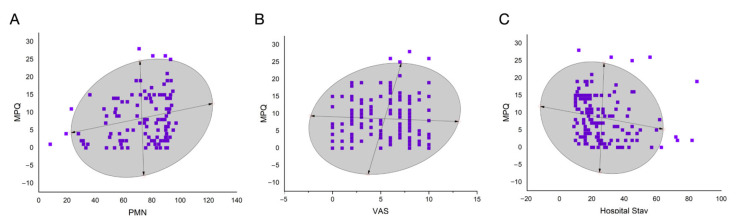
The 95% confidence ellipses for the correlation analysis. (**A**) MPQ total score and PMN%; (**B**) MPQ total score and preoperative VAS score; (**C**) MPQ total score and hospital stay.

**Figure 4:**
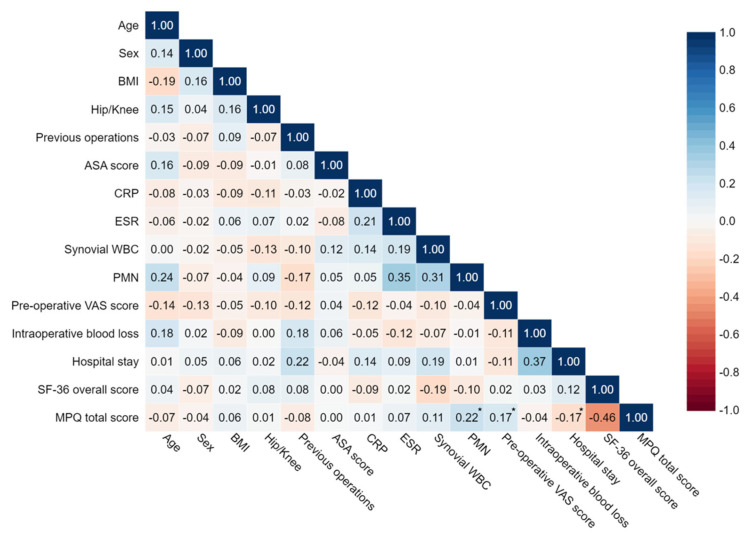
Correlation matrix of the potential risk factors for QOL and pain. (*: *p* < 0.05).

However, there was a significant positive correlation between MPQ score and PMN% (r = 0.221, 95% CI 0.063-0.362, *p* = 0.020) and preoperative VAS score (r = 0.169, 95% CI 0.003-0.323, *p* = 0.048). The hospital stay was significantly negatively correlated with the MPQ score (r = -0.168, 95% CI -0.354-0.035, *p* = 0.049). The 95% confidences ellipses are shown in Figure 3. Further linear regression analysis showed that the three indicators were potential risk factors for MPQ score [F(1, 109) = 5.604, *p* = 0.020, adjusted R square = 4%] for PMN%, [F(1, 135) = 3.979, *p* = 0.048, adjusted R square = 2.1%] for pre-operative VAS score, and [F(1, 135) = 3.936, *p* = 0.049, adjusted R square = 2.1%] or the length of hospital stay. A correlation matrix is shown in Figure 4. These data suggest that higher PMN%, higher preoperative VAS score, and shorter hospital stays lead to higher severity of pain in patients. However, it should be acknowledged that the correlation coefficient value was not high, suggesting moderate to low correlations.

## 4. Discussion

PJI is a disastrous complication after joint replacement surgery. Whether using DAIR, one-stage revision, or two-stage revision, patients usually need to undergo multiple additional major operations followed by long-term antibiotic treatment, leading to serious pain and dysfunction [9]. PJIs are the undesired result of the complex interaction of several factors according to the type of microorganism involved, the type of implanted prosthesis, and the characteristics of the patient undergoing surgery [21]. Specifically, concerning host-related factors, the immune response is known to affect the outcome of infection, which is confirmed both by the particular susceptibility to prosthetic infection of immunocompromised patients and by in vitro data on the importance of interferon-gamma (IFN-gamma) in inhibiting foreign body colonization [22]. These factors all affected the outcomes of patients with PJI.

Traditionally, surgeons often evaluate the outcomes of arthroplasty by the Harris Hip Score (HHS) for THA or the Knee Society Score (KSS) for TKA [23]. However, these indicators are usually based on the subjective judgment of clinicians. Detailed outcome assessment of how the patient perceives their status is often neglected in outcome assessments [24]. Even for primary TKA surgery, approximately 20% of patients report unsatisfactory results [25]. As such, based on objective measures, such as ligamentous stability and range of motion (ROM) to patient-reported outcome measures (PROMs), surgeons continue to evolve in determining the best way to measure ‘success’ after arthroplasty [26]. Nonetheless, studies on health-related quality of life (HRQOL) after revision arthroplasty are scarce [27].

This study was a questionnaire survey of 137 patients receiving revision surgery for PJI to explore QOL, functional recovery, and pain improvement after revision based on patient-reported outcomes. The primary finding of our study is that in our institution, the overall QOL of patients with revision surgery for PJI, whether DAIR, one-stage revision, or two-stage revision, is generally satisfactory. The average overall scores on the SF-36, PH, and MH were 70.3, 66.8, and 72.0, respectively (maximum scores of 100). 

Liu et al. reported that, one year after primary TKA and THA, the average PH score and MH score of the SF-36 were 59–74 and 70–79, respectively [17]. One year after primary hip replacement, Austin reported scores of PH and MH of approximately 50 and 60, respectively [28]. It was expected that once PJI occurs and revision surgery is performed, the QOL of patients would be significantly affected. Poulsen’s study found that patients had significantly lower health-related QOL after two-stage revision surgery than the general public [24]. Similarly, Rietbergen concluded that QOL after the two-stage revision was 12–27% lower than that of the general public [27]. Walter et al. have investigated the long-term QOL in 36 patients with achieved eradication of infection after knee PJI. Their data, with limited sample sizes, showed that PJI patients still suffer from significantly lower quality of life compared with normative data, even years after surgically successful treatment by either DAIR, one/two-stage revision exchange, or arthrodesis [12]. Our study, however, showed that patients who underwent a revision for PJI had QOL results comparable to those after primary arthroplasty. As a tertiary referral center for PJI, our institution has a high success rate. Our experience was that obtaining pathogenic data and standardized debridement techniques are the key to the success of treatment [29,30,31,32,33]. Understandably, a higher success rate means less additional surgery and a higher QOL. Furthermore, patients with DAIR had significantly higher SF-36 scores than those in the two-stage revision group. This might be explained by a more radical extensive debridement with two-stage revision than DAIR. These data also suggest that selecting the optimal surgical approach is key to patients’ pain and QOL.

The second finding of our study is that persistent pain is prevalent in patients receiving revision surgery for PJI (74.5%), but the severity was mostly mild, regardless of treatment with DAIR, one-stage revision, or two-stage revision. The average total McGill pain score was 8.6 of 45. Nevertheless, we observed six patients in the two-stage revision group reporting a VAS score of 6, which suggests moderate pain. This might be due to two radical debridement surgeries, which may cause significant soft tissue injury. However, statistical analysis did not show a significant difference in the severity of pain and pain improvement among the three groups with different surgical strategies.

Persistent pain after joint replacement is very common. It was previously reported that 8–34% of patients after TKA have pain [34]. Persistent postsurgical pain is associated with reduced HRQOL after arthroplasty [17]. Pain is more prevalent in revision surgery and more than twice as many patients had pain after revision surgery compared with patients after primary TKA [35]. While most patients have a favorable outcome after revision, some patients may continue to have significant pain [36]. However, as there is no standardized or optimal time after surgery to measure the existence of persistent pain and no standardized instrument is available to define the presence or severity of persistent pain [17], comparisons of our study to previous studies are unscientific. In our experience, to reduce the postoperative pain of patients receiving revision for PJI, a multimodal analgesia algorithm must be followed. As with primary joint replacement, we also used multimodal strategies, including preemptive analgesia, intraoperative local infiltration analgesia (LIA), and the combined use of analgesics with different pharmacology, for patients undergoing revision. On the other hand, although we do not know the specific mechanism of persistent pain after joint replacement and revision, central sensitization may be an important reason [37]. The MPQ questionnaire we incorporated in this study includes a qualitative description of the characteristics of persistent postsurgical pain, which may offer insight into mechanisms in future studies.

Third, our study demonstrated that pain was significantly positively correlated with PMN% and preoperative VAS score but was significantly negatively correlated with the length of hospital stay, despite low correlation coefficients. This is logical, because a higher proportion of neutrophils means a more serious level of local inflammation, which might also predict more severe pain after surgery. A study has revealed that PMN was able to release proinflammatory cytokines (TNF-a, IL-1b, IL-6), which in turn increased from 10- to 80-fold the intracellular expression of inducible cyclo-oxygenase (COX)-2, which are correlated not only with postoperative pain but also with poor recovery after major orthopedic interventions [38]. For primary arthroplasty, presurgical pain was the most significant clinical predictive factor [39], which is consistent with our study findings. Larsen et al.’s analysis from a larger randomized controlled trial also demonstrated high preoperative pain intensity was associated with higher chronic postoperative pain intensity at 12 months after knee and hip arthroplasty [40]. Finally, longer hospitalization also allows patients to receive systematic support from rehabilitation specialists, which may help treat pain. 

Several other risk factors for postoperative pain have been identified in the literature. Watts et al. showed that morbid obesity not only significantly increased the risk of subsequent revision, reoperation, and reinfection following two-stage revision for TKA PJI but also led to worse pain relief and overall function [41]. Another study identified medical and psychological comorbidities as risk factors for moderate to severe pain after primary and revision TKA [42]. However, we were not able to identify the association of other potential causative factors, such as the number of previous operations in the same joint or intraoperative blood loss, with QOL and pain after revision. Risk factors contributing to chronic pain after surgery remain to be investigated in future studies. Preoperative knowledge of a high probability of pain may improve risk assessment, lead to more appropriate management, and allow for time to consider alternative therapies [43].

Our study is of great clinical significance. First of all, our research shows that the QOL of patients receiving revision surgery for PJI is generally satisfactory regardless of the type of surgery the patient receives. Less invasive surgeries such as DAIR can improve the QOL. In our opinion, this is closely related to the surgical techniques of debridement and the ability of PJI diagnosis. Our previous research shows that by optimizing microbial culture methods and using new molecular diagnostic technology including metagenomic next-generation sequencing (mNGS), the microbial detection rate can be significantly improved [44,45], which can guide antibiotic treatment, improve the success rate of treatment, and ultimately improve the QOL of patients after surgery. Secondly, our research shows that despite high patient satisfaction, residual pain after a revision surgery is still prevalent. Surgeons still need to think about ways to alleviate patients’ pain, such as multimodal analgesia, change to stronger pain medication, or early rehabilitation exercise. The pathophysiological mechanism of postoperative pain also needs to be further explored. Thirdly, we found that preoperative PMN%, VAS, and length of hospital stay were significantly correlated to postoperative pain. This may prompt clinicians to pay attention to preoperative optimization. For patients commoditized with these issues before surgery, more measures should be taken to prevent postoperative pain.

This study has strengths and limitations that need to be acknowledged. It should be acknowledged that in most of the current cross-sectional studies, data were collected from many different hospitals and reflect a variety of surgical techniques and implications [17,24]. This may be argued to be a weakness in these studies, as they can be potential confounders. Moreover, some studies only reported the QOL of patients, without pain scores or other related outcome measures. Our study is a single-center study with a decent sample size. All surgeries were performed by the same experienced surgeon, which makes the results consistent. Although no previous studies are using the same measurement instrument after revision for PJI, any future studies that use this same instrument can begin to add cross-sectional and longitudinal information on QOL and persistent pain after any revision for PJI.

Our study also has several limitations. First, the response rate was relatively low, with only 64% of the patients answering the questionnaire. In particular, the sample size of patients who received one-stage revision is relatively small. However, due to the low incidence of PJI [5] and the relatively high mortality [24], the sample size can be deemed considerable. In addition, the effect sizes of some comparisons and the correlation coefficients, despite significant statistical *p*-values, were not large enough, which decreased the statistical power to draw firm conclusions. Second, this study was a cross-sectional study and lacked a series of measures at different time points after surgery. Thus, we were not able to evaluate the improvement of the patients’ QOL and pain over time. Third, this was a retrospective study, which may have resulted in recall bias. The baseline information of the included patients was relatively variable. Some patients were comorbid with different diseases, which may be confounding factors affecting QOL and pain [46]. Fourth, the questionnaires used were SF-36 and MPQ questionnaires. These questionnaires had their limitations as they focused on pain and quality of life but did not cover other critical information such as mortality. In addition, we did not use the cross-culturally adapted Chinese version of forms. For SF-36, there was one item that is translated differently than the Chinese-adapted version [47]; Item 9 (‘Walking more than a mile’) was translated as ‘Walking more than 1.61 kilometers’ but it is ‘Walking more than 1.5 km’ in the Chinese-adapted version. However, for the MPQ questionnaire, all items were the same as in the Chinese-adapted version [48]. We acknowledged that the use of self-translated questionnaires may affect the reliability, validity, and normalization of the survey. Lastly, this was a single-center study performed in a Chinese tertiary hospital. If our practice and conclusions can be generalized to other countries or races of population remains to be further clarified.

## 5. Conclusions

Our research shows that the QOL of patients receiving revision surgery for PJI is generally satisfactory and DAIR surgery has the potential in achieving higher QOL than two-stage revision. Persistent pain is prevalent in these patients, but the severity was mostly mild. There was no significant difference in the severity of pain among patients with DAIR, one-stage revision, or two-stage revision. PMN%, preoperative VAS score, and hospital stay were potential risk factors for patients’ pain after revision surgery for PJI.

## Figures and Tables

**Table 1:** Demographic information of 137 patients included in the study.

	Total	DAIR	One-Stage Revision	Two-Stage Revision	*p*-Value
Number of patients	137	42	31	64	-
Age	67.6 (9.8)	69.6 (7.2)	68.4 (9.0)	65.2 (11.0)	0.05
Sex (Male/Female)	64/73	24/18	12/19	28/36	0.24
BMI	24.1 (3.1)	24.2 (2.9)	23.9 (2.7)	24.3 (3.5)	0.96
Hip/Knee	74/63	18/24	22/9	34/30	0.06
Smoking (Yes/No)	39/98	15/27	9/22	15/49	0.39
Drinking alcohol (Yes/No)	25/112	10/32	8/23	7/57	0.11
Number of previous operations in the same joint	1.6 (0.9)	1.3 (0.5)	1.4 (0.5)	1.8 (1.1)	0.01
ASA score	2.1 (0.5)	2.1 (0.5)	1.9 (0.7)	2.1 (0.4)	0.11
Intraoperative blood loss (mL)	607.3 (550.2)	300.0 (164.7)	467.3 (233.3)	876.7 (681.0)	<0.0001
Hospital stay (days)	26.2 (15.2)	19.1 (10.1)	17.7 (6.2)	35.0 (16.4)	<0.0001
Follow-up time (months)	35.3 (20.9)	34.6 (22.8)	35.7 (20.2)	35.5 (20.3)	0.97

**Table 2:** Overall and subscale scores of SF-36 among the three groups.

	Total	DAIR	One-Stage Revision	Two-Stage Revision	*p*-Value
Number of patients	137	42	31	64	-
SF-36 overall score	70.3 (15.6)	75.2 (11.3)	70.7 (13.7)	66.7 (18.0)	0.02
Dimension A: physical health (PH)	66.8 (18.3)	71.7 (14.4)	68.6 (17.3)	62.8 (20.2)	0.04
Dimension B: mental health (MH)	72.0 (16.5)	76.6 (10.1)	71.0 (16.1)	69.4 (19.3)	0.08
Scale 1: physical functioning (PF)	62.8 (22.2)	68.6 (17.6)	68.4 (19.8)	56.3 (24.4)	0.005
Scale 2: role limitations due to physical health	55.7 (41.1)	69.3 (36.6)	54.6 (38.1)	47.3 (43.4)	0.02
Scale 3: role limitations due to emotional problems	76.3 (33.4)	84.3 (21.6)	69.2 (32.2)	74.5 (39.3)	0.13
Scale 4: energy/fatigue	69.9 (19.2)	72.9 (14.6)	70.6 (22.1)	67.5 (20.3)	0.36
Scale 5: emotional well-being	70.1 (16.8)	73.8 (15.0)	72.1 (15.8)	67.3 (17.8)	0.09
Scale 6: social functioning (SF)	77.8 (20.7)	83.9 (14.2)	74.8 (16.1)	75.2 (25.2)	0.07
Scale 7: pain	80.0 (14.7)	79.1 (11.4)	80.7 (12.6)	80.3 (17.5)	0.88
Scale 8: general health (GH)	65.8 (21.2)	68.7 (19.0)	68.5 (21.0)	62.5 (22.4)	0.24
Health change	73.9 (21.1)	77.3 (20.2)	77.8 (14.6)	69.9 (23.6)	0.11

**Table 3:** Total and subscale scores of McGill Pain Score among the three groups.

	Total	DAIR	One-Stage Revision	Two-Stage Revision	*p*-Value
Number of patients	137	42	31	64	-
Total McGill Pain Score	8.6 (6.5)	8.9 (6.4)	8.7 (5.4)	8.3 (7.1)	0.87
Sensory subscore	6.2 (5.1)	6.3 (5.3)	6.0 (4.5)	6.2 (5.4)	0.97
Affective subscore	2.4 (2.3)	2.6 (2.0)	2.7 (2.3)	2.0 (2.5)	0.28
VAS	1.8 (1.6)	1.7 (1.4)	1.4 (1.3)	2.1 (1.9)	0.09
Present pain intensity (PPI)	0.7 (0.6)	0.7 (0.6)	0.5 (0.5)	0.8 (0.7)	0.25

**Table 4:** Potential risk factors for QOL and pain after revision surgery for PJI.

		SF-36 Overall Score			MPQ Total Score	
	Pearson Correlation (r)	95% CI	*p*-Value	Pearson Correlation (r)	95% CI	*p*-Value
Age	0.040	−0.162–0.274	0.646	−0.072	−0.267–0.107	0.402
Sex	−0.066	−0.232–0.098	0.441	−0.036	−0.198–0.134	0.674
BMI	0.024	−0.140–0.173	0.801	0.062	−0.118–0.269	0.508
Hip/Knee	0.081	−0.105–0.253	0.347	0.015	−0.149–0.177	0.864
Number of previous operations in the same joint	0.077	−0.053–0.223	0.374	−0.077	−0.262–0.113	0.372
ASA score	0.002	−0.151–0.154	0.982	0.004	−0.136–0.151	0.960
CRP	−0.088	−0.278–0.104	0.312	0.010	−0.152–0.169	0.912
ESR	0.015	−0.149–0.191	0.866	0.065	−0.128–0.240	0.463
Synovial fluid WBC	−0.187	−0.356–−0.034	0.050	0.114	−0.083–0.362	0.235
PMN%	−0.099	−0.264–0.079	0.299	0.221	0.063–0.362	0.020 ^a^
Pre-operative VAS score	0.016	−0.147–0.186	0.855	0.169	0.003–0.323	0.048 ^a^
Intraoperative blood loss	0.027	−0.142–0.211	0.758	−0.036	−0.245–0.168	0.678
Hospital stay	0.118	−0.021–0.250	0.170	−0.168	−0.354–0.035	0.049 ^a^

^a^ The correlation is significant at a level of 0.05 (two-tailed).

## Data Availability

The data presented in this study are available in this article and its Supplementary Materials.

## Supplementary Materials

The following supporting information can be downloaded at: https://www.mdpi.com/article/10.3390/jcm11237182/s1. Supplementary File S1: Questionnaire.
