# Supplementary material for: Patient-Reported Outcome on Quality of Life and Pain after Revision Arthroplasty for Periprosthetic Joint Infection: A Cross-Sectional Study
Source: J Clin Med. 2022 Dec 2;11(23):7182. doi: 10.3390/jcm11237182 (PMC9741318; doi:10.3390/jcm11237182)
Supplement: Supplementary file 1 [file jcm-11-07182-s001.zip › jcm-2054495-supplementary.pdf]

Supplementary Materials. Survey questionnaire

Section A. SF-36 Questionnaire

Choose one option for each questionnaire item.

1. In general, would you say your health is:

（1）Excellent
（2）Very good
（3）Good
（4）Fair
（5）Poor

2. Compared to one year ago, how would you rate your health in general now?

（1）Much better than one year ago
(2) Somewhat better than one year ago
(3) About the same
(4) Somewhat worse than one year ago
(5) Much worse than one year ago

The following items are about activities you might do during a typical day. Does your health now limit you in these activities? If so, how much?

3. Vigorous activities, such as running, lifting heavy objects, participating in strenuous sports.

（1）Yes, limited a lot
（2）Yes, limited a little
（3）No, not limited at all

4. Moderate activities, such as moving a table, pushing a vacuum cleaner, bowling, or playing golf

（1）Yes, limited a lot
（2）Yes, limited a little
（3）No, not limited at all

5. Lifting or carrying groceries

（1）Yes, limited a lot
（2）Yes, limited a little
（3）No, not limited at all

6. Climbing several flights of stairs.

（1）Yes, limited a lot
（2）Yes, limited a little
（3）No, not limited at all

.

7. Climbing one flight of stairs

（1）Yes, limited a lot
（2）Yes, limited a little
（3）No, not limited at all

8. Bending, kneeling, or stooping

（1）Yes, limited a lot
（2）Yes, limited a little
（3）No, not limited at all

9. Walking more than a mile

（1）Yes, limited a lot
（2）Yes, limited a little
（3）No, not limited at all

10. Walking several blocks

（1）Yes, limited a lot
（2）Yes, limited a little
（3）No, not limited at all

11. Walking one block

（1）Yes, limited a lot
（2）Yes, limited a little
（3）No, not limited at all

12. Bathing or dressing yourself.

（1）Yes, limited a lot
（2）Yes, limited a little
（3）No, not limited at all

work or other regular daily activities as a result of your physical health?

13.Cut down the amount of time you spent on work or other activities.
（1）Yes
（2）No

14. Accomplished less than you would like
（1）Yes
（2）No

15. Were limited in the kind of work or other activities.
（1）Yes
（2）No

16. Had difficulty performing the work or other activities (for example, it took extra effort).
（1）Yes
（2）No

During the past 4 weeks, have you had any of the following problems with your work or other regular daily activities as a result of any emotional problems (such as feeling depressed or anxious)?

17.Cut down the amount of time you spent on work or other activities.
（1）Yes
（2）No

18. Accomplished less than you would like.
（1）Yes
（2）No

19. Didn't do work or other activities as carefully as usual.
（1）Yes
（2）No

20. During the past 4 weeks, to what extent has your physical health or emotional problems interfered with your normal social activities with family, friends, neighbors, or groups?
（1）Not at all
（2）Slightly
（3）Moderately
（4）Quite a bit
（5）Extremely

21. How much pain have you had during the past 4 weeks?
（1）None
（2）Very mild
（3）Mild
（4）Moderate
（5）Severe
（6）Very severe

22. During the past 4 weeks, how much did pain interfere with your normal work (including both work outside the home and housework)?
（1）Not at all
（2）Slightly
（3）Moderately
（4）Quite a bit
（5）Extremely

These questions are about how you feel and how things have been with you during the past 4 weeks. For each question, please give the one answer that comes closest to the way you have been feeling.

How much of the time during the past 4 weeks.

23. Did you feel full of pep?
（1）All of the time

（2）Most of the time
（3）A good bit of the time
（4）Some of the time
（5）A little of the time
（6）None of the time

24. Have you been a nervous person?
（1）All of the time
（2）Most of the time
（3）A good bit of the time
（4）Some of the time
（5）A little of the time
（6）None of the time

25. Have you felt so down in the dumps that nothing could cheer your up?
（1）All of the time
（2）Most of the time
（3）A good bit of the time
（4）Some of the time
（5）A little of the time
（6）None of the time

26. Have you felt calm and peaceful?
（1）All of the time
（2）Most of the time
（3）A good bit of the time
（4）Some of the time
（5）A little of the time
（6）None of the time

27. Did you have a lot of energy?
（1）All of the time
（2）Most of the time
（3）A good bit of the time
（4）Some of the time
（5）A little of the time
（6）None of the time

28. Have you felt downhearted and blue?
（1）All of the time
（2）Most of the time
（3）A good bit of the time
（4）Some of the time
（5）A little of the time
（6）None of the time

29. Did you feel worn out?
（1）All of the time
（2）Most of the time
（3）A good bit of the time
（4）Some of the time
（5）A little of the time
（6）None of the time

30. Have you been a happy person?
（1）All of the time
（2）Most of the time
（3）A good bit of the time
（4）Some of the time
（5）A little of the time
（6）None of the time

31. Did you feel tired?
（1）All of the time
（2）Most of the time
（3）A good bit of the time
（4）Some of the time
（5）A little of the time
（6）None of the time

32. During the past 4 weeks, how much of the time has your physical health or emotional problems interfered with your social activities (like visiting with friends, relatives, etc.)?
（1）All of the time
（2）Most of the time
（3）A good bit of the time
（4）Some of the time
（5）A little of the time
（6）None of the time

How TRUE or FALSE is each of the following statements for you.

33.I seem to get sick a little easier than other people.

（1）Definitely true
（2）Mostly true
（3）Don’t know
（4）Mostly false
（5）Definitely false

34. I am as healthy as anybody I know

（1）Definitely true
（2）Mostly true
（3）Don’t know
（4）Mostly false
（5）Definitely false

35. I expect my health to get worse

（1）Definitely true
（2）Mostly true
（3）Don’t know
（4）Mostly false
（5）Definitely false
（5）Definitely false

36. My health is excellent

（1）Definitely true
（2）Mostly true
（3）Don’t know
（4）Mostly false
（5）Definitely false

Section B. McGill Pain Questionnaire

SENSORY Dimension of Pain Experience with the replaced joint (Questions 1-11)

1. Throbbing
（0）None
（1）Mild
（3）Moderate
（4）Severe

2. Shooting
（0）None
（1）Mild
（3）Moderate
（4）Severe

3. Stabbing
（0）None
（1）Mild
（3）Moderate
（4）Severe

4. Sharp
（0）None
（1）Mild
（3）Moderate
（4）Severe

5. Cramping
（0）None
（1）Mild
（3）Moderate
（4）Severe

6. Gnawing
（0）None
（1）Mild
（3）Moderate
（4）Severe

7. Hot-burning
（0）None
（1）Mild
（3）Moderate
（4）Severe

8. Aching
（0）None
（1）Mild
（3）Moderate
（4）Severe

9. Heavy
（0）None
（1）Mild
（3）Moderate
（4）Severe

10. Tender
（0）None
（1）Mild
（3）Moderate
（4）Severe

11. Splitting
（0）None
（1）Mild
（3）Moderate
（4）Severe

AFFECTIVE Dimension of Pain Experience with the replaced joint (Questions12-15)

12. Tiring-exhausting
（0）None
（1）Mild
（3）Moderate
（4）Severe

13. Sickening
（0）None
（1）Mild
（3）Moderate
（4）Severe

14. Fearful
（0）None

（1）Mild
（3）Moderate
（4）Severe

15. Punishing-cruel
（0）None
（1）Mild
（3）Moderate
（4）Severe

Visual Analogue Scale (Pain Severity):
Please evaluate the severity of your pain (0 for ‘no pain’ to 10 for ‘most severe pain’).

No pain Most severe pain
0 1 2 3 4 5 6 7 8 9 1 0

Present Pain Intensity
（0）No pain
（1）Mild
（2）Discomforting
（3）Distressing
（4）Horrible
（5）Excruciating
